# The Impact of Differential Parenting: Study Protocol on a Longitudinal Study Investigating Child and Parent Factors on Children’s Psychosocial Health in Hong Kong

**DOI:** 10.3389/fpsyg.2020.01656

**Published:** 2020-07-28

**Authors:** Catalina Sau Man Ng, Ming Ming Chiu, Qing Zhou, Gail Heyman

**Affiliations:** ^1^Department of Early Childhood Education, The Education University of Hong Kong, Hong Kong, China; ^2^Department of Special Education and Counselling, The Education University of Hong Kong, Hong Kong, China; ^3^Department of Psychology, University of California, Berkeley, Berkeley, CA, United States; ^4^Department of Psychology, University of California, San Diego, San Diego, CA, United States

**Keywords:** differential parenting, psychosocial health and well-being, empathy, perception of fairness, personality, Chinese parents, adolescents, longitudinal study

## Abstract

Adolescents who believe that their parents treat them differently from their siblings have poorer psychosocial well-being than otherwise. This phenomenon, which is known as parental differential treatment or PDT occurs in up to 65% of families. Past studies have examined socio-demographic variables (e.g., child gender, age, and birth order) as predictors of PDT, but these immutable characteristics do little to inform interventions and help these adolescents. Hence, this study extends past research by investigating links among parent empathy, parent perception of PDT, child perception of PDT, child perception of fairness and child well-being (self-esteem, depression, anxiety, and trust in the relationship with parents). Furthermore, this study tests whether adolescent personality (openness, conscientiousness, extraversion, agreeableness, and neuroticism), child empathy, and child perception of fairness moderate these links. This study will utilize a two-wave longitudinal design with a 1-year lapse. Data will be collected from 760 Chinese adolescents studying from Secondary One to Secondary Three in 18 schools in Hong Kong and from their parents. We test our theoretical model via a multilevel structural equation model (ML-SEM). This study both addresses (a) theoretical debates about relations among empathy, PDT, fairness, and psychosocial well-being and (b) focuses on modifiable factors and behaviors, to inform future interventions, such as parent education.

## Introduction

Mental health conditions account for 16% of illnesses and injuries in 10- to 19-year-olds (World Health Organization, 2018), so adolescent psychosocial health is a growing global public health issue (Patel et al., 2007). Adolescents with poor psychosocial well-being are more likely than others to experience adverse health and developmental outcomes, such as nicotine addiction, alcohol abuse, substance abuse, school failure and dropout, delinquent behaviors, teenage pregnancy, self-harm, or suicide (Kessler et al., 1997; Dani and Harris, 2005; Ng, 2007; Lalayants and Prince, 2014; Hjorth et al., 2016; Thompson et al., 2018). Failing to address adolescent psychosocial well-being not only impairs both physical and mental health but also yields adverse effects extending into adulthood (Kansky et al., 2016), contributing to unhappy and unfulfilling lives (World Health Organization, 2018). Therefore, studying adolescent psychosocial health can generate knowledge that helps adolescents develop into healthy adults.

Parenting practices can influence children’s psychosocial well-being (Boyle et al., 2004). As parents might adapt their parenting to each of their children’s personality (Ceresnie, 2015) or needs, some children perceive that “parental behaviors are being directed unequally toward them and their siblings” [*parental differential treatment* (PDT), Stocker et al., 1997]. Indeed, studies suggest that PDT is prevalent in many families (65% in the United States, Brody et al., 1998; 63.3% in Belgium, Jeannin and Van Leeuwen, 2015).

Children who perceive greater PDT than other children may feel competition or injustice among siblings, with both favored and disfavored children showing poorer mental health (Shanahan et al., 2008; Pillemer et al., 2010). Furthermore, PDT is linked to indicators of adolescent well-being, including self-esteem, anxiety, suicidal ideation, and delinquency (Conger and Conger, 1994; Sheehan and Noller, 2002; De Man et al., 2003). When adolescent children were disfavored over time, externalizing behaviors increased (Richmond et al., 2005). These behaviors include aggression and internalizing symptoms, including anxiety, depression, and poor adjustment (Kowal et al., 2002). Children deprived of parental warmth were more likely than other children to show depression symptoms, which were more prevalent among (a) girls than boys and (b) older children than younger children (Shanahan et al., 2008). In a monozygotic-twin differences study, children perceiving greater differences in parental coldness than other children showed more internalizing disorders (Long et al., 2015). Moreover, children who perceived more differential parenting than others were more likely to show adult psychopathology (Long et al., 2015), poorer self-esteem, or less trust in relationships (Guerrero, 1998; Volling et al., 1998; Rauer and Volling, 2007).

Past cross-sectional studies in Western societies showed that several immutable characteristics were linked to PDT: family structure, family size, birth order, and gender. Specifically, single parents and larger families showed more PDT than other families did (Jenkins et al., 2003). Also, parents often favored the youngest child over other children (Rohde et al., 2003). As mothers viewed daughters as sources of emotional and instrumental support, mothers often favored daughters over sons (Suitor and Pillemer, 2006). However, few studies examined the links between PDT and child or parent psychological characteristics, which interventions might modify (unlike demographic factors).

### Relationships Among Child and Parental Factors, PDT, and Child Psychosocial Health

**FIGURE 1:**
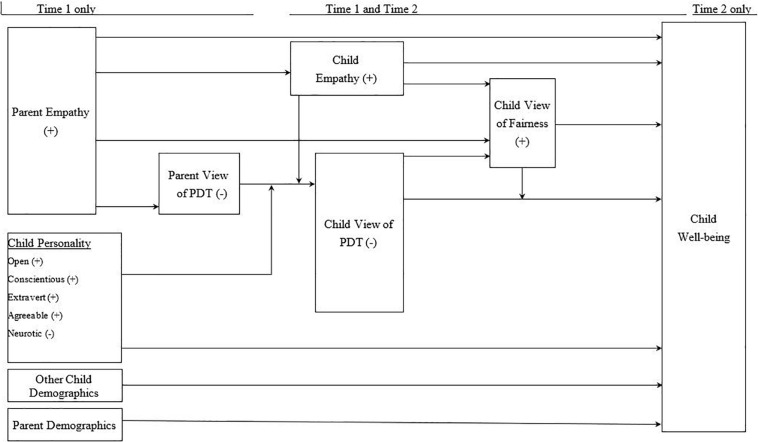
A hypothesized model of the effects of perceived differential parental treatment, child empathy and personality, child’s perception of fairness, and parental empathy on the child’s psychosocial well-being.

Although past studies have linked PDT and adolescent psychosocial health, they have not fully documented its antecedents, mediators, or moderators (Loeser et al., 2016). Hence, we proposed and empirically test the following theoretical model (see Figure 1) regarding personality, empathy, PDT, fairness, and psychosocial well-being.

### Theoretical Background Linking Parental Differential Treatment to Adolescent Well-Being

Individuals often evaluate themselves via comparisons with others (*social comparison theory*, Festinger, 1954), and these comparisons are often associated with their self-esteem (Feinberg et al., 2000). Whereas comparisons to those with less favorable experiences or performances (*downward comparisons*) raise a person’s self-esteem, upward comparisons decrease self-esteem (Mendes et al., 2001). Furthermore, children with lower self-esteem than others compare themselves with their siblings more often (Feinberg et al., 2000). Children may use PDT to infer how their parents evaluate them and might internalize these evaluations. Unfavorable comparisons with siblings can yield negative self-evaluations, which can lead to more anxiety, depressive symptoms, and problem behaviors (e.g., using tobacco, alcohol, truancy; McHale et al., 1995; Kowal et al., 2002; Shanahan et al., 2008; Loeser et al., 2016). Furthermore, disfavored children often develop trust issues and resentment, and as they get older, these feelings may have a negative impact on other relationships (Heinonen et al., 2005).

#### Fairness

Fairness might mediate or moderate the link between PDT and psychological well-being. *Fairness* refers to the degree to which people judge whether resources are fairly shared among themselves based on their belief in (a) their deserved portion and (b) their received portion (*distributive justice framework*, Deutsch, 1985). When they believe their received portion is unfair, they may show negative behaviors (Deutsch, 1985). Hence, a child who judges PDT as unwarranted and hence unfair may be less respectful of their parents and react more negatively to parental discipline, which can harm the parent–child relationship, reduce parental support, and damage a child’s psychosocial well-being.

Fairness can also moderate the link of PDT to psychosocial well-being. The perception of PDT as fair (e.g., due to an ill sibling’s greater needs or a caring sibling’s frequent help) might weaken the negative link between PDT and psychosocial well-being (McHale and Pawletko, 1992; Kowal and Kramer, 1997; McHale et al., 2000; Kowal et al., 2002; Jensen and McHale, 2017). Hence, we hypothesize that perception of fairness both mediates and moderates the link between PDT and psychosocial well-being.

#### Empathy

A child’s empathy might affect his or her perceived PDT, fairness, or psychological well-being. Empathy is “the trait or tendencies of a person to both affectively experience emotions of concern at the suffering of others and to cognitively adopt the perspective of another” (Manczak et al., 2016, p. 211). Empathy is assessed in terms of children’s understanding of their parents’ emotions and behaviors, which may help them make sense of PDT, and judge the same level of PDT to be fairer or at least more reasonable. Therefore, children who are relatively high in empathy are likely to have fewer grievances against their parents and have fewer conflicts with parents – all of which can enhance their parental relationships and their psychosocial well-being. Thus, we propose that high levels of empathy are associated with (a) lower levels of PDT, (b) higher levels of perceived fairness, and (c) higher levels of well-being.

When parents have a high level of empathy, it might enhance their children’s psychological well-being, in part via (a) greater child empathy, (b) less PDT, or (c) greater child perception of fairness. Notably, parents with more empathy are less likely to physically abuse their children than other parents (Feshbach, 1987). Empathetic parents can show greater warmth and caring for their children, interact harmoniously with them, share greater positive affect, and foster greater reciprocal responsiveness than parents who are low in empathy (Kochanska, 1997). Hence, parents with higher empathy might engage in less PDT than those with lower empathy (e.g., Leerkes, 2010; Ewing et al., 2019). Furthermore, empathetic parents might explain the rationale for their PDT, so that their children judge it to be fairer than other children do. As a result, children with more empathetic parents than others might show greater empathy, less PDT, more perception of fairness, fewer internalizing problems (e.g., anxiety, depression, distrust), and fewer externalizing problems (e.g., aggression, Eisenberg et al., 1991; Strayer and Roberts, 2004).

#### Child Personality

Child personality (neuroticism, agreeableness, openness) might be linked to PDT and psychological well-being (e.g., VanderZee et al., 1996). As people with greater neuroticism than others are upset more easily, they are more sensitive to PDT (McCullough et al., 2001), which can amplify or perpetuate negative emotions (Matthews et al., 2006), such as anxiety or depression (Griffith et al., 2010). In contrast, adolescents who are more agreeable than others might tolerate or accept others’ behaviors more readily, which might weaken their perception of PDT and enhance their psychosocial well-being. Meanwhile, adolescents with greater openness than others might be more willing to listen to parental explanations of PDT, understand them, and appreciate them, thereby weakening their perception of PDT and enhancing their psychosocial well-being. In short, child neuroticism, agreeableness, and openness might influence both PDT and psychosocial well-being.

### The Current Study

Unlike past cross-sectional studies of immutable characteristics in Western societies that cannot test the directionality of relations (Meunier et al., 2012), the present longitudinal study (1) examines the prevalence of PDT in Hong Kong; (2) tests whether the link between PDT and adolescent psychosocial well-being is mediated and moderated by adolescent perception of fairness, and moderated by adolescent personality, parent empathy or adolescent empathy; and (3) tests whether PDT at baseline (Time One) affects adolescent psychosocial well-being at follow-up (Time Two).

To our knowledge, only one published cross-sectional study investigated PDT in Hong Kong (De Man et al., 2003); it showed that perceived parental disfavor had the strongest link to suicidal ideation among Chinese adolescents. Compared to Western parents, Chinese parents are more likely to use threats, apply psychological control, emphasize hard work, and encourage harmony in family relationships (Chen and Uttal, 1988; Shek, 2001; Olsen et al., 2002). Thus, Hong Kong provides a unique cultural context to examine PDT and its links to adolescent adjustment.

## Methods

### Study Design

The study aims to examine whether PDT affected adolescent psychosocial well-being via a two-wave longitudinal, quantitative survey. Past studies showed that adolescents are more sensitive than younger children to PDT, so we studied adolescents aged 12–14 years (Daniels and Plomin, 1985). Given the intensity of changes in personal relationships during adolescence (Santrock, 1996), the effects of PDT can emerge more consistently if measured across a year (Shebloski et al., 2005), so we use a 1-year lapse between waves 1 and 2.

### Sample Size and Statistical Power

Based on past studies showing a non-significant intraclass correlation (Richmond et al., 2005), the power analysis software G^∗^Power 3 (Version 3.1) estimates that a sample size of 547 participants is adequate to detect a small effect size of 0.12 with 80% statistical power at α = 0.05 and two waves of data (Richmond et al., 2005). Owing to the possibility of missing data (i.e., 20%) (Brody et al., 1992; Shebloski et al., 2005), the expected sample size for the baseline data of the proposed study is 684. For wave 2, we assume an attrition rate of 10% for a school-based study (Brook et al., 2010), so the minimum sample size required for the proposed study is 760. Thus, we recruited at least 760 Chinese students and their parents from local secondary schools to meet the targeted sample size. Simulations of structural equation models show that this sample size exceeds the minimal sample needed (440), even for little explained variance (Wolf et al., 2013).

### Selection of Schools

In Hong Kong, 393 schools are mostly funded by the government and follow the government curriculum (government or aided schools), while 60 schools receive less government funding and have more curriculum flexibility (Direct Subsidy Scheme). Thus, we selected 15 of the former and 3 of the latter (18 schools from 18 school districts). For each consenting school, one class was randomly selected from each of three grades (secondary one to secondary three, equivalent to grades 7–9) for a total of 54 classes (= 3 classes × 18 schools).

### Participants

The inclusion criteria for adolescents and their parents are listed below.

(a) Adolescents: Chinese adolescents studying junior secondary schools (i.e., secondary one to secondary three; equivalent to grades 7–9) were invited to join the study, with the exclusion of those clinically diagnosed with cognitive or learning problems.
(b) Parents: (1) Chinese parents of children in grades 7–9 (both fathers and mothers; if the family was non-intact or either the father or mother passed away, the parent who lived with the adolescent completed the questionnaire; (2) have at least two children; and (3) can read Chinese.

### Data Collection

#### Procedures of Data Collection at Baseline: Schools

To recruit a school from each of the 18 districts, we first prepared a list of schools in each district based on the information from the Education Bureau website. To recruit the schools, we first emailed an invitation letter. We followed up with calls to the principals to explain the objectives of the proposed study and the data collection procedures. If the school declined to join the study, another school of the same type in the same district was invited.

#### Procedures of Data Collection at Baseline: Parents

After schools agreed to join the study, we sent a letter explaining our study to parents or guardians (for youth under age 18) of the participating secondary students. Parents were asked to (a) give written consent to join the study and allow their child to participate or (b) decline. Collecting both “yes” and “no” reply forms enabled us to measure the extent to which all parents were given the letter of invitation by their children. Consenting parents then received the questionnaire and instructions in a sealed envelope, via their children. On the questionnaire, we stated the anonymity and confidentiality of data and their right to withdraw from the study at any time without any consequences. Both parents were instructed to complete the questionnaire independently and place it in the self-adhesive envelope previously supplied with the questionnaire. Their children brought the completed questionnaires to us via their school. Participating parents and children stated the last four numerical numbers of the children’s Hong Kong Identity Card (HKID) and date of birth to match the student and parent data. The odds of even a single coincidental match is less than 1% (if needed, we can also check child gender and family structure).

#### Procedures of Data Collection at Baseline: Students

Consenting adolescents completed the questionnaire in a class period of 40 min at school in the presence of a trained research assistant. Students were reminded of their data’s anonymity and confidentiality, and their right to withdraw from the study at any time without any consequences. To avoid excluding ineligible students from their classmates’ shared activity, all students completed the questionnaires, and those of ineligible participants were destroyed. We followed all ethical procedures for research set by the University Human Research Ethics Committee.

#### Follow-Up (T_2_) (1-Year Interval)

The same procedure will be used for the follow-up data collection with parents and children.

## Measurements

Questionnaires for parents and children differ except for the PDT and empathy sections.

### Parent Questionnaire

#### Sociodemographic Information

Gender, age, marital status, education level, occupation, monthly income, district of residence, total number of children, date of birth of each child.

#### Parental Differential Treatment

Parents were asked to complete the Sibling Inventory of Differential Experience (SIDE) (Daniels and Plomin, 1985). The parent version of the SIDE assesses differential affection and differential control. The Differential Affection subscale has five items that focus on parents’ differential pride, enjoyment, understanding, interest, and favoritism. The Differential Control subscale has four items and measures parents’ differential strictness, punishment, discipline, and blame. Parents will rate the one among their children who is closest in age to the student participant. Items were scored on a 5-point Likert scale to assess whether specific behaviors occurred: 1 = Much more with the older child, 2 = A bit more with the older child, 3 = Same for both children, 4 = A bit more with the younger child, or 5 = Much more with the younger child. The Cronbach’s alpha coefficient was 0.94 for the mother and 0.95 for the father (Young, 2011).

#### Parental Empathy

To measure empathetic response, we used two subscales of the Chinese Interpersonal Reactivity Index (C-IRI, Siu and Shek, 2005), specifically Perspective Taking (e.g., “Before criticizing somebody, I try to imagine how I would feel if I were in their place”) and Empathetic Concern (e.g., “I am often quite touched by things that I see happen”). Participants rated each item on a 5-point scale from 0 = “does not describe me well” to 4 = “describes me very well.” The Cronbach’s alpha was 0.78 for perspective taking and 0.75 for empathetic concern, respectively (Li et al., 2019).

### Student Questionnaire

Students completed a questionnaire including the following items:

#### Sociodemographic Information

Date of birth, gender, grade level in school, sibling position in family.

#### Perceived Parental Differential Treatment

The nine items of the SIDE assess PDT (Daniels and Plomin, 1985). This scale has two dimensions: Differential Parental Affection (five items) and Differential Parental Control (four items). Participants rated each item on a 5-point Likert scale to assess their perception of how differently their parents treated their closest-age sibling versus themselves based on his or her experience over the past 12 months. For example, my father/mother “has enjoyed doing things with us” (Affection scale) or “has punished us for our misbehavior” (Control scale). The respondents chose an answer by selecting one option (1 = My sibling a lot more, 2 = My sibling a little more, 3 = Treat both of us equally, 4 = Me a little more, 5 = Me a lot more).

Regarding the prevalence of PDT in Hong Kong (objective 1), we will report the degrees of perceived PDT as perceived by both types of study participants: (a) parents and (b) their adolescent children.

Respondents assessed parental treatment separately for each parent, yielding four measures (i.e., Differential Paternal Affection, Differential Paternal Control, Differential Maternal Affection, Differential Maternal Control). De Man et al. (2003) used this scale in a local study on PDT and suicidal ideation in Hong Kong. The internal consistency was 0.76 for the Affection scale and 0.81 for the Control scale (Kowal et al., 2004).

#### Child Personality

The NEO Five-Factor Inventory (NEO-FFI) measures personality and consists of five 12-item scales, one for each personality dimension (openness, conscientiousness, extraversion, agreeableness, and neuroticism, Costa and McCrae, 1992). Each item is rated on a 5-point scale, ranging from 0 to 4. NEO-FFI has been used in Hong Kong (e.g., Leung et al., 2013). An example item of each dimension is “is outgoing, sociable” (extraversion); “is helpful and unselfish with others” (agreeableness), “perseveres until the task is finished” (conscientiousness), “is depressed, blue” (neuroticism). The internal consistency ranged from 0.69 to 0.81 (Leung et al., 2013).

#### Child Empathy

As the C-IRI (Siu and Shek, 2005) scale is also suitable for adolescents, we used it to measure child empathy. The Cronbach’s alpha was 0.79 for perspective taking and 0.63 for empathetic concern, respectively (Au et al., 2011).

#### Child Perception of Fairness of PDT

Respondents were asked to rate the fairness of each SIDE (Sibling Inventory of Differential Experience) item. Participants’ responses to the Control and Affection items of the SIDE will be coded as 0 = “unfair” or 1 = fair (Kowal et al., 2002).

#### Depression

The Chinese version of the Center for Epidemiologic Studies Depression Scale for Children (CES-DC) measured children’s depressive symptoms (psychological well-being). There are 20 items with each item rated on a 4-point scale ranging from 0 (“rarely or none of the time”) to 3 (“most or all of the time”). Example items include, “I felt depressed” and “I had trouble keeping my mind on what I was doing.” The summary score ranged from 0 to 60. Cronbach’s alpha was 0.82 in the study of Li et al. (2010).

#### Anxiety

The seven-item anxiety subscale of the Depression Anxiety Stress Scale (DASS) assessed symptoms of anxiety (Lovibond and Lovibond, 1995). The scores have been categorized into different levels: normal, mild, moderate, severe, and extremely severe. Wang et al. (2016) used the Chinese version of the DASS in clinical and non-clinical settings, and it showed good validity and reliability (α = 0.80).

#### Self-Esteem

The Chinese version of Rosenberg’s Self-Esteem Scale (C-RSES) measured respondents’ self-esteem. The scale has 10 items, and participants rated items on a 5-point scale, ranging from 1 (“strongly disagree”) to 5 (“strongly agree”) with higher scores indicating higher levels of self-esteem. Example items include, “On the whole, I am satisfied with myself.” The Cronbach’s alpha of C-RSES was 0.88 in the study of Hong et al. (2020).

#### Trust in Relationships

Six self-constructed items were used to measure the levels of trust in parent-child relationships on a 4-point scale with 1 being “never” to 4 “Always” (e.g., “My father trusts me.”, “My mother trusts my judgment.”). The Cronbach’s alphas of the pilot study for mother and father were 0.85 and 0.63, respectively.

## Proposed Analysis

### Analytic Issues and Statistics Strategies

Suitable analyses of these data must address the issues described below involving data, outcomes, and explanatory variables (see Table 1). Data issues include missing data and survey measurement error. As missing data can bias results, reduce estimation efficiency, or complicate data analyses, we estimate the missing data with Markov Chain Monte Carlo multiple imputation, which outperforms listwise deletion, pairwise deletion, mean substitution, and simple imputation according to computer simulations (Peugh and Enders, 2004).

**TABLE 1:** Statistics strategies to address each analytic difficulty.

**Analytic difficulty**	**Statistics strategy**
**Data set**	
• Missing data	• Markov Chain Monte Carlo multiple imputation (Peugh and Enders, 2004)
• Measurement errors on surveys	• Factor Analysis (Joreskog and Sorbom, 2018) • Item Response model (Embretson and Reise, 2013)
**Outcome variables**	
• Nested data (students in classes in schools)	• Multilevel analysis (aka Hierarchical linear modeling, Goldstein, 2011)
• Multiple outcomes (Y_1_, **Y_2, …_**)	• Structural equation model (Joreskog and Sorbom, 2018)
**Explanatory variables**	
• Indirect, mediation effects (X →**M**→Y)	• Structural equation model (Joreskog and Sorbom, 2018)
• Interaction in structural equation model	• Residual centering (Crandall et al., 2012)
• Many hypotheses’ false positives	• Two-stage linear step-up procedure (Benjamini et al., 2006)
• Compare effect sizes (β_1_ > β_2_?)	• Lagrange multiplier tests (Bertsekas, 2014)
• Consistency of results across data sets (Robustness)	• Separate single outcome models • Original (not estimated) data

To minimize survey measurement error, we use multiple questions for each construct to create a precise index. We analyze whether sets of questions reflect one or more underlying constructs (e.g., depression) through factor analyses (Joreskog and Sorbom, 2018).

Outcome issues include nested data and multiple outcomes. As students in the same classroom and school have more shared experiences and likely resemble one another more than those in different classrooms or schools (nested data), an ordinary least squares regression underestimates the standard errors, so we use a multilevel analysis (Goldstein, 2011; also known as hierarchical linear modeling, Bryk and Raudenbush, 1992). Also, multiple outcomes can have correlated residuals that underestimate standard errors, which we address via a multilevel structural equation model (ML-SEM, Joreskog and Sorbom, 2018).

Explanatory variable issues include mediation effects, moderation effects, many hypotheses’ false positives, effect size comparisons, and robustness. Separate, single-level tests of mediation effects on nested data can bias results, so we test for simultaneous mediation effects with an ML-SEM (Crandall et al., 2012). As moderation (interaction) terms are often correlated with their component variables and can yield unstable results, we use residual centering to remove such correlations before testing for moderation effects (Crandall et al., 2012). As testing many hypotheses increases the possibility of a false positive, we reduce its likelihood via the two-stage linear step-up procedure, which outperformed 13 other methods in computer simulations (Benjamini et al., 2006).

When testing whether the effect sizes of explanatory variables differ, Wald and likelihood ratio tests do not apply at boundary points. Hence, we use Lagrange multiplier tests, which apply to the entire data set and show greater statistical power than Wald or likelihood ratio tests for small deviations from the null hypothesis (Bertsekas, 2014).

Lastly, we test whether the results remain stable despite minor changes in the data or analyses (robustness, Kennedy, 2008). As a misspecified equation for any outcome in a multivariate outcome model can introduce errors in otherwise correctly specified equations, we model each outcome variable separately. Next, we run subsets of the data separately. Then, we repeat the analyses for the original, unestimated data.

### Factor Analyses

We test the internal validity of the survey items for each construct and minimize their measurement errors with confirmatory factor analyses (CFA, Joreskog and Sorbom, 2018). To assess the fit of the CFA, we use the comparative fit index (CFI), Tucker–Lewis index (TLI), standardized root mean square residual (SRMR), and root mean square error approximation (RMSEA), which minimize type I and type II errors under many conditions in Hu and Bentler’s (1999) simulations. Fit thresholds are as follows: good (CFI and TLI > 0.95; SRMR < 0.08; RMSEA < 0.06), moderate (0.90 < CFI and TLI < 0.95; 0.08 < SRMR < 0.10; 0.06 < RMSEA < 0.10), and poor (CFI and TLI < 0.90; SRMR > 0.10; RMSEA > 0.10).

### Explanatory Model

We test our mediation and moderation models (objective 2) and determine whether child experience of PDT at time 1 predicts well-being at time 2 (objective 3) via an ML-SEM with LISREL 10.1 (Joreskog and Sorbom, 2018) as follows:

$$W\,e\,l\,l\,\text{-}\,B\,e\,i\,n\,g_{i\,j\,k}=B\,P\,a\,r\,e\,n\,t\,\_\,D\,e\,m\,o\,g\,r\,a\,p\,h\,i\,c\,s_{i\,j\,k}+$$

$$\Gamma \,C\,h\,i\,l\,d\,\_\,D\,e\,m\,o\,g\,r\,a\,p\,h\,i\,c\,s_{i\,j\,k}$$

$$+\vartheta \,P\,a\,r\,e\,n\,t\,\_\,E\,m\,p\,a\,t\,h\,y_{i\,j\,k}+\Phi \,P\,a\,r\,e\,n\,t\,\_\,P\,D\,T_{i\,j\,k}$$

$$+\Theta \,C\,h\,i\,l\,d\,\_\,E\,m\,p\,a\,t\,h\,y_{i\,j\,k}+\Omega \,C\,h\,i\,l\,d\,\_\,p\,e\,r\,c\,e\,i\,v\,e\,d\,\_\,P\,D\,T_{i\,j\,k}$$

$$+\sigma \,C\,h\,i\,l\,d\,\_\,p\,e\,r\,c\,e\,i\,v\,e\,d\,\_\,f\,a\,i\,r\,n\,e\,s\,s_{i\,j\,k}+\Psi \,I\,n\,t\,e\,r\,a\,c\,t\,i\,o\,n\,s$$

(1) $$+v+\eta_{k}+\delta_{j\,k}+\varepsilon_{i\,j\,k}$$

Well-Being outcomes are depression, anxiety, self-esteem, and trust in relationships. The sets of explanatory variables of Parent_Demographics, Child_Demographics, Parent_Empathy, Parent_PDT, Child_Empathy, Child_perceived_PDT*_*i*_*, and Child_perceived_fairness have corresponding sets of parameters: *B*, Γ, *ϑ*, Φ, Θ, Ω, σ, and Ψ. Meanwhile, **ν** is a vector of intercepts, and the unexplained components (residuals) at the school, classroom, and individual levels are **η***_*k*_*, **δ***_*jk*_*, and ***ε****_*i*_*, respectively.

First, we enter the fixed, structural demographics of each parent (mother_age, mother_marital_status, mother_education, mother_job, mother_monthly_income, mother_district_of_residence, mother_total_children, father_ age, father_marital_status, father_education, father_job, father_monthly_income, father_district_of_residence, father_ total_children) and of the child (gender, age, younger sisters, younger brothers, older sisters, older brothers). These demographics might affect parent processes. As parent empathy might affect parent PDT, we enter them in that order (mother empathy, father empathy, mother PDT, father PDT). Parent processes might affect child processes. Child empathy might influence child-perceived PDT, which in turn might influence child-perceived fairness, so we enter them in that order. Then, we test for specific moderation effects: (a) whether parent PDT interactions with child personality (neurotic, agreeable, open) or child empathy are linked to child-perceived PDT and (b) whether child PDT interaction with child-perceived fairness are linked to child well-being.

## Time Frame for the Study

The study started in January 2019 and will last for 26 months. The data collection at Time One has already started. The whole study will be completed in February 2021 (see Table 2).

**TABLE 2:** Timeline for data collection.

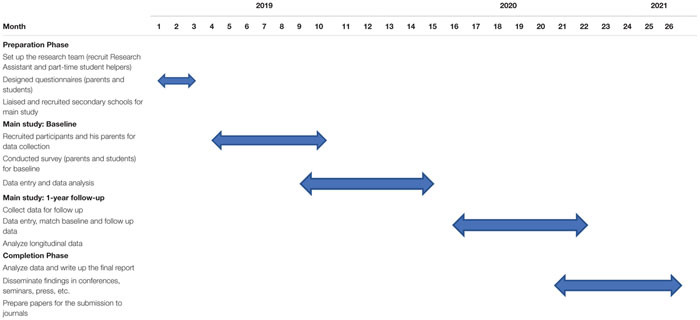

## Discussion

The two-wave study will expand our knowledge of links between PDT and adolescent psychosocial well-being by testing links among parent empathy, parent-perceived PDT, child-perceived PDT, child-perceived fairness, and child well-being. Furthermore, we test whether child personality, child empathy, or child-perceived fairness moderate the above links. It is the first study, to our knowledge, to test whether higher levels of child and parental empathy affect child’s perceived fairness of PDT, which might protect the psychosocial well-being of adolescents. As this study examines modifiable psychological processes, its results can inform the development of family- and parent-based interventions to reduce PDT and support adolescent psychosocial well-being.

The present study has a number of strengths. First, by focusing on modifiable rather than unchangeable factors and behaviors predicting PDT, the study results can inform future family based interventions. The proposed research is a pioneering study that will both inform efforts to promote adolescent psychosocial well-being in Hong Kong and address important theoretical debates about the relations among parent empathy, child empathy, PDT, child perception of fairness, and psychosocial well-being. Furthermore, the study uses multiple informants’ reports, including those of parents and children, and it will also yield findings from a large sample of 18 secondary schools from the 18 districts of Hong Kong. Also, we test our theoretical model with multi-informant data from a large sample and with advanced statistics strategies that address many analytic concerns (e.g., multilevel structural equation model).

## Limitations

Similar to other studies, this study has some limitations. First, the sample included only Chinese adolescents in Hong Kong, which limits the generalizability of the results. Second, the study used self-reported questionnaires so there is a risk that the participants may provide socially desirable answers. Therefore, including qualitative interviews to triangulate the findings is needed. Third, the present study includes only two-wave data collection, which may not adequately assess changes. Therefore, at least three waves should be included in future studies to sufficiently describe trajectories of variables and covariates over time.

## Conclusion

This study is among the first to examine PDT in Asia and is the first to investigate the prevalence of PDT in Hong Kong. The results will improve our understanding of PDT, inform interventions to improve the psychosocial well-being of adolescents, and stimulate more comparative studies between Western and East Asian societies. These comparisons are particularly important given the 2015 shift from a one-child policy to a two-child policy in mainland China.

## Data Availability Statement

The datasets generated for this study are available on request to the corresponding author.

## Ethics Statement

The studies involving human participants were reviewed and approved by the Human Research Ethics Committee (HREC) of The Education University of Hong Kong approved the ethical approval for this study (Reference Number: 2017-2018-0192). Written informed consent to participate in this study was provided by the participants and their legal guardian/next of kin where appropriate.

## Author Contributions

CN is the principal investigator of the study and she is primarily responsible for the original design and development of the study, and prepared the first draft of this study protocol. MC and QZ contributed to the study design. CN and MC prepared the initial proposal for funding application. QZ provided methodological expertise in the study design. CN, MC, GH, and QZ contributed to the rewriting and refinements. All the authors have read and approved the final version of the manuscript.

## Conflict of Interest

The authors declare that the research was conducted in the absence of any commercial or financial relationships that could be construed as a potential conflict of interest.
